# Genetic Connectivity between North and South Mid-Atlantic Ridge Chemosynthetic Bivalves and Their Symbionts

**DOI:** 10.1371/journal.pone.0039994

**Published:** 2012-07-06

**Authors:** Karina van der Heijden, Jillian M. Petersen, Nicole Dubilier, Christian Borowski

**Affiliations:** Symbiosis Group, Max Planck Institute for Marine Microbiology, Bremen, Germany; University of Canterbury, New Zealand

## Abstract

Transform faults are geological structures that interrupt the continuity of mid-ocean ridges and can act as dispersal barriers for hydrothermal vent organisms. In the equatorial Atlantic Ocean, it has been hypothesized that long transform faults impede gene flow between the northern and the southern Mid-Atlantic Ridge (MAR) and disconnect a northern from a southern biogeographic province. To test if there is a barrier effect in the equatorial Atlantic, we examined phylogenetic relationships of chemosynthetic bivalves and their bacterial symbionts from the recently discovered southern MAR hydrothermal vents at 5°S and 9°S. We examined *Bathymodiolus* spp. mussels and *Abyssogena southwardae* clams using the mitochondrial cytochrome c oxidase subunit I (COI) gene as a phylogenetic marker for the hosts and the bacterial 16S rRNA gene as a marker for the symbionts. *Bathymodiolus* spp. from the two southern sites were genetically divergent from the northern MAR species *B. azoricus* and *B. puteoserpentis* but all four host lineages form a monophyletic group indicating that they radiated after divergence from their northern Atlantic sister group, the *B. boomerang* species complex. This suggests dispersal of *Bathymodiolus* species from north to south across the equatorial belt. 16S rRNA genealogies of chemoautotrophic and methanotrophic symbionts of *Bathymodiolus* spp. were inconsistent and did not match the host COI genealogy indicating disconnected biogeography patterns. The vesicomyid clam *Abyssogena southwardae* from 5°S shared an identical COI haplotype with *A. southwardae* from the Logatchev vent field on the northern MAR and their symbionts shared identical 16S phylotypes, suggesting gene flow across the Equator. Our results indicate genetic connectivity between the northern and southern MAR and suggest that a strict dispersal barrier does not exist.

## Introduction

Fracture zones on the ocean floor dissect the mid-oceanic ridges causing trench-like transform faults. They disconnect adjacent ridge segments by lateral offsets of tens to hundreds of kilometers and are thought to be geological barriers for the dispersal of hydrothermal vent organisms, thus affecting the biogeography of hydrothermal vent communities [1]. Hydrothermal vent species disperse predominantly as larvae in the water column. They rise up with the hydrothermal plume, migrate passively with currents along the ridge axes and colonize other vent sites downstream [2], [3]. Transform faults may impede species dispersal along the mid-ocean ridges by the loss of larvae from the terminal ends of ridge segments into the open ocean [4], [5]. The segregating effect of single topographic seafloor structures on species distribution has been tested recently by examining gene flow between closely related populations along ridge systems. For example, the Blanco Transform Fault in the eastern Pacific separating Juan de Fuca Ridge (JdF) and Gorda Ridge isolates mitochondrial gene haplotypes of morphologically similar limpets of the genus *Lepetodrilus* [6], alvinellid polychaetes display limited gene flow across fracture zones on the East Pacific Rise (EPR) [7], [8], and the Easter Island Microplate on the EPR isolates the mussel species *Bathymodiolus thermophilus* and *B*. aff. *thermophilus* [9]. In contrast, each of the two species *“Calyptogena” magnifica* and *B. thermophilus* share similar haplotypes along more than 4500 kilometers and across several transform faults on the EPR (*“C.” magnifica*: 21°N to 17°S) [10] or the EPR and the Galapagos Rift (*B. thermophilus*: 13°N to 17°S and Rose Garden) [9], indicating that species with sufficient dispersal potential can bridge pronounced ridge discontinuities.

**Figure 1:**
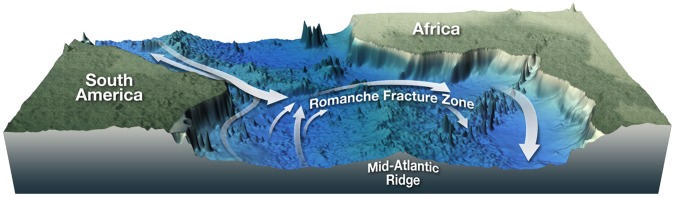
Deep-water currents are channeled through the equatorial transform faults crossing the Mid Atlantic Ridge. According to current hypotheses, these currents build a conduit for dispersal of chemosynthetic organisms across the Atlantic, and together with ridge-axis offset and transform fault topography they may hinder dispersal of larvae from north to south on the MAR (after [72]).

In the equatorial Atlantic Ocean, a number of large transform faults interrupt the Mid-Atlantic Ridge (MAR). The largest is the Romanche Transform Fault, which reaches 7370 m depth [11] and creates an offset of 935 km in the ridge axis. These deep valleys channel Atlantic deep water across the MAR from West to East [1], [12] (Fig. 1), and it has been hypothesized that they affect the biogeography of chemosynthetic communities in two major ways. Firstly, it was suggested that the effects of large ridge offset, pronounced trench topography, and currents through the transform faults impede the dispersal of vent invertebrates between the northern and southern Mid-Atlantic Ridge (NMAR and SMAR) [13], [14]. Hydrothermal vents on the SMAR were unknown until recently and hypotheses on the barrier effect included the prospect that the equatorial belt might separate two distinct biogeographic MAR provinces, and that SMAR province communities might be most similar to Indian Ridge communities [1]. Secondly, it was hypothesized that the equatorial across-axis currents provide a west-east passage for chemosynthetic species and that the transform faults act as conduits to gene-flow across the MAR [1], [13] resulting in very similar seep communities on both sides of the Atlantic. Recent studies provide evidence for such a passage [15], [16].

**Figure 2:**
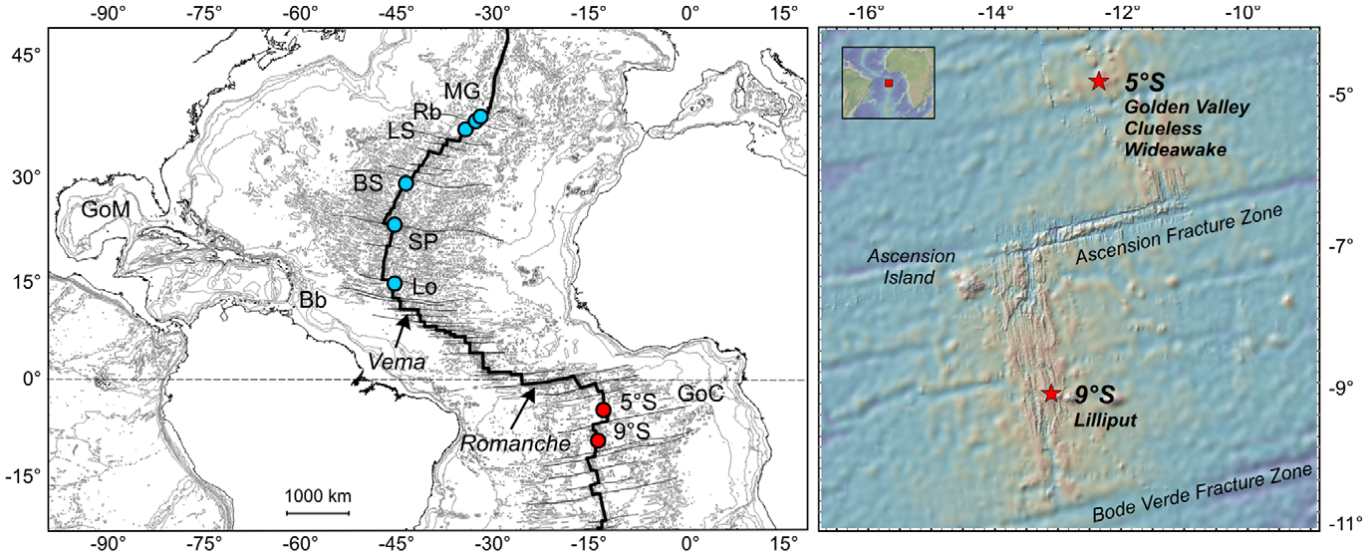
Hydrothermal vents on the MAR with chemosynthetic bivalves and hydrocarbon seep areas on the western and eastern Atlantic margins with the *B. boomerang* species complex. Left: Overview on North Atlantic vents and seeps. Vema and Romanche are large transform faults. Right: Locations of the 5°S and 9°S vent fields on the MAR. 5°S = SMAR vents Golden Valley, Clueless and Wideawake; 9°S = SMAR vent field Lilliput; Bb = Barbados Accretionary Prism; BS = Broken Spur; GoC = Gulf of Congo; GoM = Gulf of Mexico, Lo = Logatchev Hydrothermal Vent Field; LS = Lucky Strike; MG = Menez Gwen; Rb = Rainbow; SP = Snake Pit.

The discovery of the hydrothermal vents and their chemosynthetic communities at 5°S and 9°S on the MAR in 2005 [17], [18], [19] (Fig. 2) provided the first opportunity to test if the Romanche Transform Fault inhibits hydrothermal vent species dispersal between NMAR and SMAR and divides these two regions into separate biogeographic provinces. The 5°S vents are located in 3000 m water depth on a 2 km long volcanically active plateau of the ridge axis and include three high-temperature vents and numerous low-temperature vent sites with diffuse fluid flow [18]. The 9°S vents lie in 1500 m water depth and consist of several low-temperature diffuse flow sites in an area of 300 m by 400 m, while hot vents have not been found [19]. Both vent fields harbor chemosynthetic communities that at first view resemble those of the NMAR. *Bathymodiolus* spp. are dominant at sites with diffuse venting. The mussels at 5°S are morphologically similar to the NMAR species *B. puteoserpentis* while 9°S mussels display more variable shell morphology and appear more similar to *B. azoricus* (R. von Cosel, pers. information) although their average body size was much smaller, as it was observed during repeated visits within several years [19]. Alvinocarid *Rimicaris* shrimp colonize the sulfide edifices of hot venting at 5°S. A vesicomyid clam found at 5°S was identified as *Abyssogena southwardae* based on its morphology [20], a species known from the Logatchev vent field on the NMAR [20], [21]. Vestimentiferan tubeworms typical for EPR vents or provannid gastropods characteristic for western Pacific and Indian Ridge vents were not found [18], [19].

In this study, we investigated the phylogenetic relationships of the chemosynthetic bivalves and their associated symbiotic bacteria from the new SMAR vent fields. We tested whether the close relationships of *Bathymodiolus* spp. and *Abyssogena southwardae* to their northern relatives based on morphology is supported by molecular data and if gene flow may have occurred across the equatorial belt. We also tested if the genetic data reveal phylogenetic relationships to chemosynthetic symbioses from the Pacific or Indian Oceans. We used the mitochondrial cytochrome c oxidase subunit I (COI) as a marker gene for the host species and 16S rRNA for their bacterial symbionts.

## Materials and Methods

### Animal Collection

**Figure 3:**
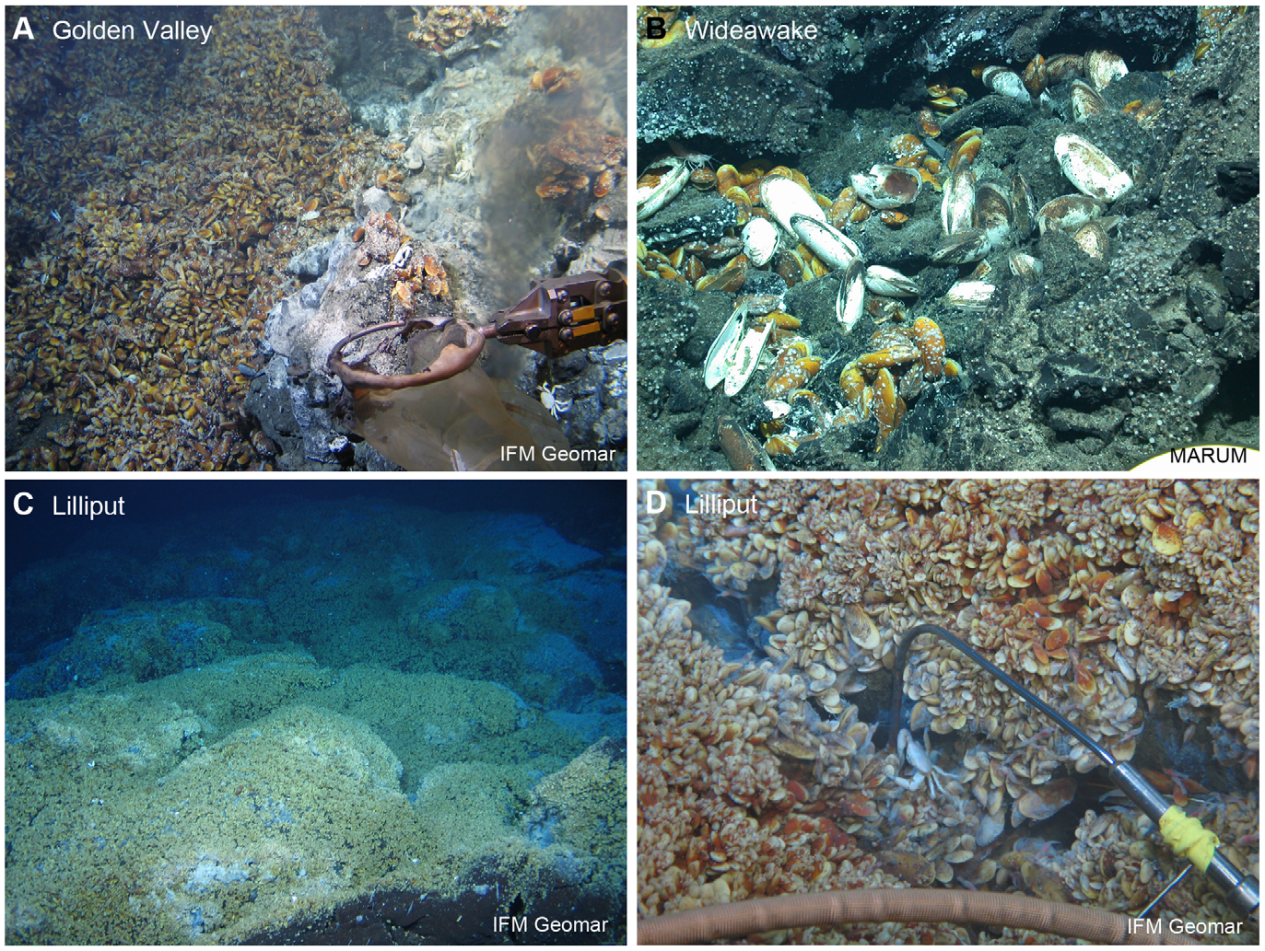
Sampling locations in the SMAR vents. A: The Golden Valley vent covers approximately 30 m of a long N-S oriented 3–5 m deep fissure at 4°48.16′S, 12°22.28′W. The walls and the bottom of the fissure are densely covered with golden-brown *Bathymodiolus* sp. which gave rise to the name. B: The Wideawake vent site lies in a jumbled lava sheet flow and is characterized by extensive beds of *Bathymodiolus* sp. and associated diverse fauna, including occasional *Abyssogena southwardae* clams. C: Small *Bathymodiolus* sp. covering pillow lava and semi-lithified Fe-oxyhydroxide crust at the Lilliput vent field at 9°32.83′S, 13°37.74′S. D: Close-up of Lilliput mussels.

Animal material used in this study was collected on the SMAR during RV Meteor research cruises M64/1 in May 2005 and M78/2 in May 2009 and RV l’Atalante cruise MARSUED IV in January 2008 using the ROVs Marum Quest 4000 m (Marum, Bremen) and Kiel 6000 (IFM-GEOMAR, Kiel) (Table 1). Additional NMAR material was sampled in the Logatchev hydrothermal vent field in January 2007 using ROV Jason II (Woods Hole Oceanographic Institution) during Maria S. Merian cruise MSM 04/3. For the 5°S area, 32 specimens of *Bathymodiolus* sp. obtained from three different diffuse vent sites were used for genetic analyses. The vent sites named Golden Valley (n = 10 specimens), Clueless (n = 10) and Wideawake (n = 12) were about 850 m from each other in 2987–2998 m water depth (Fig. 3A and B). Two *Abyssogena* specimens were collected from the Clueless vent site at 5°S. For the 9°S area 16 *Bathymodiolus* sp. specimens were used from the Lilliput vent field in 1595 m water depth (Fig. 3C and D). 21 specimens of *B. puteoserpentis* were obtained from the Irina II mussel bed at Logatchev in 3020 m water depth. Onboard, all specimens were kept in chilled seawater and processed as soon as possible with a maximum of 12 hours after recovery. Animals were dissected and gill tissue samples for DNA extraction were stored at −20°C until further processing.

**Table 1:** Material, sampling coordinates and accession numbers of investigated specimens and published sequences which were used for distance calculations (Table 2) and the reconstruction of the COI Neighbor Joining tree (Fig. 4).

Host species	Sample	Individual ID	Sampling location	Coordinates	Depth (m)	Acc COI	Acc 16S SOX	Acc 16S MOX	Ref.
*B. puteoserpentis*	MSM04/3 244 Rov 9	30, 32, 34–38, 41–44, 46,48, 49, 53–59	Logatchev, Irina II	14°45.180′ N, 44°58.746′ W	3022	JQ844834 - JQ844854	-	-	This study
*Bathymodiolus* sp. 5°S	M78/2 274 ROV 3	1, **2**, 4–11	Golden Valley	4°48.162′ S, 12°21.680′ W	2987	JQ844798 - JQ844807	-	-	This study
*Bathymodiolus* sp. 5°S	M78/2 302 ROV 15	1, 2, 3–8, 10, 11	Clueless	4°48.250′ S, 12°22.259′ W	2995	JQ844808 - JQ844817	JQ844767 - JQ844771	-	This study
*Bathymodiolus* sp. 5°S	ATA 37 Rov 7	9–11, 13–19	Wideawake	04°48.626′ S, 12°22.342′ W	2998	JQ844788 - JQ844797	-	-	This study
*Bathymodiolus* sp. 5°S	M64/1 109 GTV-A	**7**	Wideawake	4°29.184′ S, 12°13.416′ W	2998	-	JQ844772 - JQ844774	-	This study
*Bathymodiolus* sp. 5°S	M64/1 125 Rov 7	**4**	Wideawake	4°48.62′ S,12°22.35′ W	2987	-	-	JQ844775	This study
*Bathymodiolus* sp. 9°S	M78/2 319 ROV 10	1–6, **7**, 8, **9**, 10, 11, 26,31, 32, 36	Lilliput	9°32.837′ S, 13°12.549′ W	1495	JQ844818–JQ844832	JQ844777JQ844778JQ844780JQ844781	JQ844776 JQ844779	This study
*Bathymodiolus* sp. 9°S	M64/1 200 ROV 9	**4**	Lilliput	9°19.716′ S, 13°07.536′ W	1495	JQ844833	JQ844783 -JQ844785	JQ844782	This study
*B. azoricus*	n.d.	n.d.	Rainbow	36°13′ N–33°54′ W	2500	FJ766849–FJ766857,FJ766870	-	-	[40]
*B. heckerae*	n.d.	n.d.	West Florida Escarpment	26°01.8′ N,84°54.9′ W	3314	AY649794	-	-	[73]
*B. boomerang*	n.d.	n.d.	Barbados (Orenoque Dome B)	10°19.9′ N,58°37.3′ W	1950	DQ513448	-	-	[15]
*B.* aff. *boomerang*	n.d.	n.d.	Gulf of Congo	5°47.8′S, 9°42.7′ E	3170	DQ513450	-	-	[15]
*A. southwardae* 5°S	ATA 52 ROV 11	**1, 2**	Clueless	4°28.8′ S,12°22.2′ W	2995	JQ844786, JQ844787	JQ844761 - JQ844766	-	This study
*A. southwardae* Logatchev	n.d.	n.d.	Logatchev	14°45.189′ N,44°58.829′ W;14°45.32′ N,44–58.79′ W	3028–3038	AF114401–AF114403	EU403435	-	[33], [60]
*A. southwardae* GoM	n.d.	n.d.	West Florida Escarpment	26°01.8′ N,84°54.6′ W	3313	AF008280	-	-	[44]
*A. southwardae* Barbados	n.d.	n.d.	BarbadosAccretionaryPrism	13°50′ N, 57°45′ W	5000	AF008279	-	-	[44]

Individuals used for clone libraries of the bacterial 16S rRNA gene in bold; SOX = sulfur oxidizers; MOX = methane oxidizers, n.d. = no data.

### DNA Extraction, PCR Amplification, Cloning and Sequencing

Genomic DNA was extracted from mussel and clam gill tissues using the DNeasy blood and tissue kit (QIAGEN, Hilden, Germany) according to the kit manual. DNA was stored in aliquots at −20°C and later used for amplification of COI genes of the host species and 16S rRNA genes of the bacterial gill symbionts.

Partial sequences (ranging from 550 to 591 nucleotides) of the COI of *Bathymodiolus* specimens were amplified using the primers BathCOI-F (5′-TGTGGTCTGGAATAATTGGAAC-3′) and BathCOI-R (5-ATAAAAAGATGTATTRAARTGACG-3′) [15]. The DNA amplification conditions were the same as described in Olu-Le Roy et al. (2007) [15]. Mitochondrial COI of the vesicomyid clam specimens was amplified using the primers LCO1490 (5′-GGTCAACAAATCATAAAGATATTGG-3′) and HCO2198 (5′ TAAACTTCAGGGTGACCAAAAAATCA-3′) [22]. PCR conditions were as described in Petersen et al. (2010) [23]. PCR products were purified with the QIAquick PCR Purification Kit (QIAGEN, Hilden, Germany) using the microcentrifuge protocol. Purified PCR products served for direct sequencing of COI using an ABI PRISM 3100 genetic analyzer (Applied Biosystems, Foster City, CA, USA) with ABI BigDye and the same primers as in the amplification reaction.

16S rRNA genes of bacterial symbionts were amplified from three *Bathymodiolus* specimens collected at Wideawake and Clueless at 5°S, three *Bathymodiolus* specimens from Lilliput at 9°S and two *Abyssogena* specimens from 5°S using the universal bacterial primers 8F (5′-AGAGGTTGATCMTGGC-3′) and 1492R (5′-TACCTTFTTACGACTT-3′) [24]. PCR products were checked by gel electrophoresis and bands of 1500 nucleotides (nt) length were isolated and purified with the QIAquick Gel Extraction Kit (QIAGEN, Hilden, Germany). Clean PCR products were cloned to separate co-occurring phylotypes in the genomic DNA. PCR amplification, cloning and sequencing of 16S rRNA genes followed the protocol in Petersen et al. (2010) [23]. All sequences are deposited at Genbank under the accession numbers provided in Table 1.

### Phylogenetic Analyses

Mitochondrial COI sequences of *Bathymodiolus* and *Abyssogena* specimens were assembled using the Sequencher program (http://www.genecodes.com), imported into the ARB software package [25] and aligned against published sequences of bathymodiolin and vesicomyid species.

Pairwise genetic distances between COI sequences of *Bathymodiolus* from SMAR and Logatchev and published sequences of *B. azoricus* and the closest relatives to NMAR species from the western and eastern Atlantic margins were calculated for 519 nt based on the Kimura 2-parameter (K2P) model using MEGA version 5 [26]. K2P distances of *Abyssogena* spp. from 5°S, Logatchev, Gulf of Mexico (GoM) and Barbados were calculated for 513 nt. The K2P model was chosen because it allows for higher probability of transitional vs. transversional base substitution and has been used in earlier phylogenetic studies on bathymodiolins [9], [27]. Differentiation of *Bathymodiolus* populations was tested based on a Markov Chain method with the nonparametric exact test by Raymond and Rousset (1995) [28] using Arlequin software ver. 3.5.1.2 [29]. Nucleotide diversity within *Bathymodiolus* populations was calculated using DnaSP ver 5.10.01 [30]. Synonymy of *Bathymodiolus* COI gene nucleotide substitution was checked according to the standard invertebrate translation code (MEGA5) starting with nt 3 in the 519 nt alignment.

Phylogenetic analyses of bathymodiolins and vesicomyids were performed with the ARB software package [25] using 372 nt for bathymodiolins and 513 nt for vesicomyids. Maximum Likelihood (ML) phylogenies were calculated using the PhyML algorithm with the Generalized Time Reversible (GTR) nucleotide substitution model that was determined as the best fitting model by MODELTEST software [31]. Ratios of transitions and transversions, proportion of invariable sites and base frequencies were estimated empirically by ARB software. Bootstrapping used 1000 re-samplings.

Bacterial 16S rRNA genes were first analyzed based on partial clone sequences covering approx. 900 nt. The sequences were aligned against close relatives in ARB using the SILVA small subunit alignment [32]. Representative sequences of the major clone sequence groups were chosen for full length sequencing of the 16S rRNA gene (resulting sequences with 1323–1506 nt). 1208 nt of the full length sequences were used for further phylogenetic analyses. ML phylogenies were calculated with PhyML implemented in the ARB package using the Hasegawa, Kishino and Yano (HKY) nucleotide substitution model which is widely used for bacterial 16S rRNA analyses and allows for different base frequencies. Ratios of transitions and transversions, proportion of invariable sites and base frequencies were estimated empirically by ARB. Bootstrapping used 1000 re-samplings.

## Results

### Mitochondrial COI Sequence Analyses of the Hosts

#### Bathymodiolus *spp*

**Figure 4:**
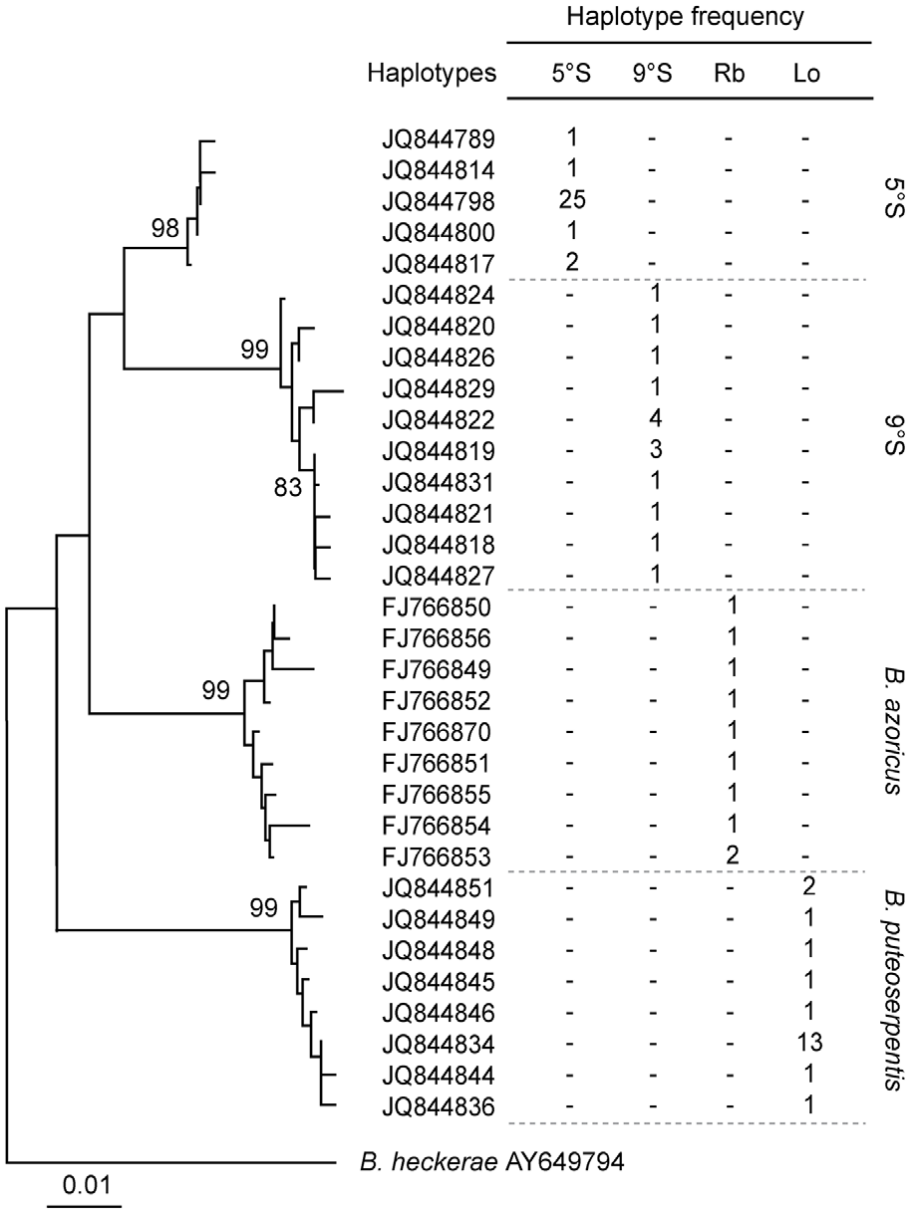
Neighbor Joining tree based on the K2P substitution model and frequencies of COI haplotypes of *Bathymodiolus* spp. from SMAR and NMAR vent fields and *B. heckerae* as an outgroup (calculated with MEGA5 [**26**]). RB = Rainbow, Lo = Logatchev. Bootstrap percentage values (1000 replicates) higher 70% are marked for the relevant branches. Scale bar represents 1% estimated base substitution.

The COI sequence analysis revealed two clearly separated lineages of *Bathymodiolus* from 5°S and 9°S which diverged from each other as well as from the NMAR species *B. azoricus* and *B. puteoserpentis* (Fig. 4). There was no geographic overlap of haplotypes among the four lineages and differences between all of them were confirmed by the exact test for population differentiation (*B. azoricus* vs. 9°S p<0.014, all other p values <0.001). In 30 specimens from the three sampling sites at 5°S, we found a total of five haplotypes of which the dominant one occurred in 25 individuals (Fig. 4). This group of haplotypes included 4 polymorphic sites; the nucleotide diversity Pi was 0.00308. 15 analyzed COI sequences from the 9°S sampling site revealed 10 haplotypes with 11 polymorphic sites (Pi = 0.00612). 21 sequences of *B. puteoserpentis* revealed 8 haplotypes of which one was strongly dominant (8 polymorphic sites; Pi = 0.0042) while 10 published sequences of *B. azoricus* were much more diverse with 9 haplotypes (12 polymorphic sites; Pi = 0.00685). The 5°S and 9°S lineages were clearly separated, with a K2P distance of 0.036 and 14 fixed nucleotide substitutions (Table 2). The divergence between the two SMAR populations based on K2P distance and numbers of fixed substitutions was only about half of the divergence between the two NMAR species (Table 2). In addition to its close relationship to 9°S, the 5°S lineage was similarly closely related to *B. azoricus* as evidenced by 16 fixed nucleotide substitutions and a genetic distance that was only slightly larger than to 9°S (Table 2). All nucleotide substitutions were synonymous among the 32 haplotypes of all MAR populations and did not lead to amino acid substitutions according to the standard invertebrate translation code. Nonsynonymous substitutions occurred in the North Atlantic seep species *B. heckerae* (M → L at codon 46 and V → D at codon 132 for *B. heckerae*) and *B*. aff. *boomerang* (D → N at codon 171).

**Table 2:** Average pairwise nucleotide divergence (K2P; mean, standard deviation in brackets) among MAR *Bathymodiolus* populations and their closest relatives from the GoM, Barbados Accretionary Prism and Gulf of Congo (GoC) (above diagonal), within populations (diagonal, italics), and the number of fixed nucleotide substitutions (below diagonal) in 519 positions of COI sequences.

	*B. azoricus*		*B. puteoserpentis*		*Bathymodiolus* sp. 5°S		*Bathymodiolus* sp. 9°S		*B. heckerae*		*B. boomerang*		*B.* aff. *boomerang*	
*B. azoricus*	*0.0064*	*(0.0035)*	0.0666	(0.0023)	0.0416	(0.0031)	0.0652	(0.0036)	0.0841	(0.0028)	0.0781	(0.0033)	0.0757	(0.0033)
*B. puteoserpentis*	26		*0.0018*	*(0.0019)*	0.0567	(0.0015)	0.0552	(0.0016)	0.0879	(0.0014)	0.0792	(0.0014)	0.0768	(0.0014)
*Bathymodiolus* sp. 5°S	16		22		*0.0006*	*(0.0010)*	0.0365	(0.0020)	0.0721	(0.0009)	0.0613	(0.0009)	0.0634	(0.0009)
*Bathymodiolus* sp. 9°S	24		21		14		*0.0046*	*(0.0028)*	0.0862	(0.0026)	0.0707	(0.0026)	0.0726	(0.0026)
*B. heckerae*	n.d.		n.d.		n.d.		n.d.		-		0.0137	(- )	0.0177	(- )
*B. boomerang*	n.d.		n.d.		n.d.		n.d.		n.d.		-		0.0078	(- )
*B.* aff. *boomerang (GoC)*	n.d.		n.d.		n.d.		n.d.		n.d.		n.d.		-	

Comparisons are based on 30 analyzed sequences for *Bathymodiolus* sp. 5°S and 15 for *Bathymodiolus* sp. 9°S, 21 analyzed sequences for *B. puteoserpentis*, 10 published sequences for *B. azoricus* [40], and single published sequences of *B. heckerae*, *B. boomerang* and *B.* aff. *boomerang* [15], [73] (n.d. = no data).

**Figure 5:**
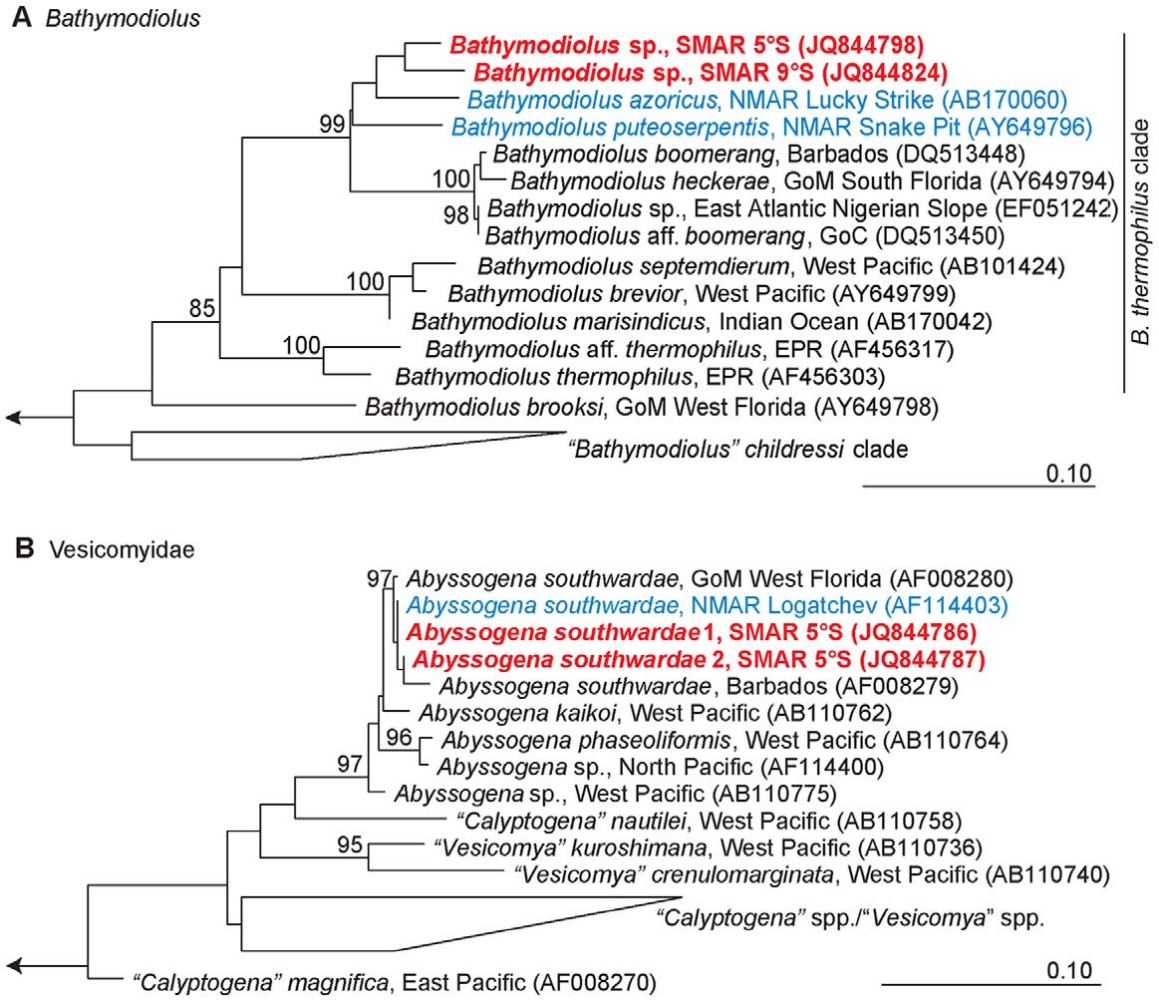
Phylogenetic reconstructions (Maximum Likelihood) based on COI. A: Bathymodiolins; B: Vesicomyidae. Large chemosynthetic vesicomyids are currently under revision and generic designations will change in the future. The notation of *Abyssogena* species in the tree follows the preliminary nomenclature of Krylova and Sahling (2010) [47] while other vesicomyid species appear under their published names. MAR species red (SMAR) and blue (NMAR). GoC = Gulf of Congo, GoM = Gulf of Mexico, EPR = East Pacific Rise. The trees were calculated with ARB software using PhyML (GTR substitution model, estimated proportion of invariable sites, four categories of substitution rates). Bootstrap values (1000 replicates) higher 70% are marked at the relevant branches. Scale bars represent 10% estimated base substitution.

Maximum Likelihood phylogenetic analysis placed the SMAR lineages in a monophyletic group with *B. azoricus* and *B. puteoserpentis* confirming the close relationship of all MAR lineages (Fig. 5A). Their status as a sister group of the North Atlantic *B. boomerang* seep species complex, which includes *B. heckerae*, *B. boomerang* and *B*. aff. *boomerang,* is well supported by bootstrap analyses. Within the MAR species, the resolution of ML was low (bootstrap support less than 55%), and repeated ML and Maximum Parsimony analyses resulted in unstable branching topologies (data not shown).

The MAR species and *B. boomerang* species complex together with a group of western Pacific and Indian Ocean species (*B. marisindicus*, *B. brevior* and *B. septemdierum*) are distinct from *B*. *thermophilus* and *B.* aff. *thermophilus* from the EPR, suggesting a common origin of species from the North Atlantic, Indian and western Pacific Oceans. However, the support for the Indian/western Pacific Ocean group was only weak (65.2%) and their positioning in this clade is uncertain.

#### Abyssogena southwardae

The two analyzed specimens from 5°S revealed two haplotypes that differed by a single nucleotide substitution. One of these was identical to one of three known haplotypes from Logatchev [33] and pairwise K2P distances between all SMAR and Logatchev haplotypes [33] did not exceed 0.0059 (Table 3, Fig. 5B). The largest divergence was measured between a Logatchev haplotype and Barbados. These data strongly suggest gene flow among *A. southwardae* populations on the MAR.

**Table 3:** Pairwise nucleotide divergence (K2P) of COI based on 513 nt among *Abyssogena southwardae* from Logatchev at the the northern Mid-Atlantic Ridge, southern Mid-Atlantic Ridge (SMAR), Florida Escarpment in the Gulf of Mexico (GoM W-Florida), and Barbados Accretionary Prism [33].

	SMAR haplotype 1	SMAR haplotype 2	Logatchev (3 haplotypes)	GoM W-Florida	Barbados Accretionary Prism
SMAR, haplotype 1	–	0.0020	0–0.0039	0.0039	0.0138
SMAR, haplotype 2		–	0.0020–0.0059	0.0059	0.0118
Logatchev, 3 haplotypes			0.0020–0.0039	0.0039–0.0076	0.0138–0.0178
GoM W-Florida				–	0.0138
Barbados Accretionary Prism					–

#### Bathymodiolus *symbionts*

16S rRNA clone libraries constructed from 6 *Bathymodiolus* specimens collected at 5°S and 9°S yielded 292 partial clone sequences that fell into two groups. These clustered with γ-proteobacterial chemoautotrophic and methanotrophic symbionts from other bathymodiolin hosts, indicating that SMAR *Bathymodiolus* spp. also live in a dual symbiosis like their NMAR relatives.

The majority (288) of the clone sequences belonged to the chemoautotrophic group. Eight clones each of the 5°S host individuals and seven from 9°S animals were selected for full-length sequencing. These 15 full-length sequences used for further analyses shared ≥99.6% sequence similarity and the two geographical groups from 5°S and 9°S differed consistently in one substitution at *E. coli* position 1029 suggesting that this substitution was site-specific. Within the 5°S group, six identical sequences originated from Wideawake and Clueless hosts while the two remaining sequences differed from these by one and three substitutions (0.08–0.25% divergence). The 9°S group included four identical sequences and three other phylotypes differing by one or two substitutions (0.08–0.17%). All of these additional substitutions occurred uniquely and in moderately to highly conserved regions of the 16S rRNA gene alignment and were not specific to geographical sites. It is therefore unclear whether they were real or due to PCR or sequencing error. In contrast, the consistent presence of a site-specific substitution in all analyzed sequences suggested that *Bathymodiolus* hosts in 5°S and 9°S harbor very similar but distinct phylotypes of chemoautotrophic symbionts. A comparison with the chemoautotrophic symbiont of *B. azoricus* [34] revealed that the dominant 5°S phylotype differed by 13 substitutions (1.0% divergence) and the 9°S phylotype by 14 substitutions (1.2%).

**Figure 6:**
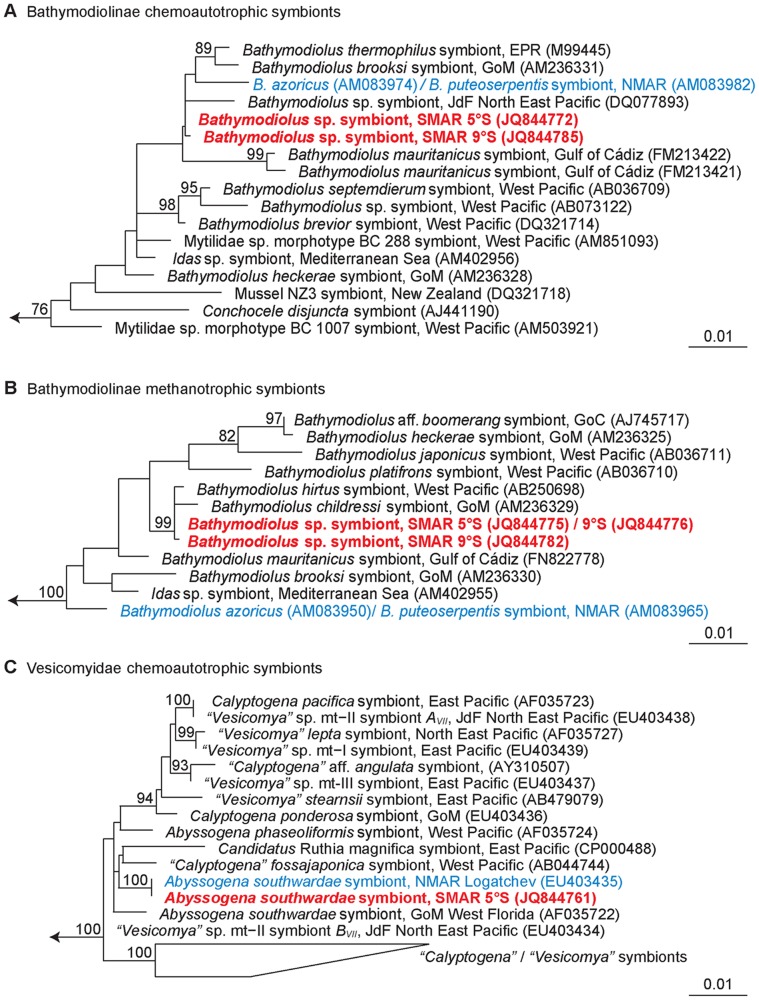
Reconstruction of symbiont phylogenies (Maximum Likelihood) based on the 16S rRNA gene. A: Chemoautotrophic symbionts of *Bathymodiolus* spp. B: Methanotrophic symbionts of *Bathymodiolus* spp. C: Chemoautotrophic symbionts of Vesicomyidae. For the notation of vesicomyid host species names, see legend for Figure 4. MAR species red (SMAR) and blue (NMAR). GoC = Gulf of Congo, JdF = Juan de Fuca Ridge. The trees were calculated with ARB software using PhyML (HKY substitution model, estimated proportion of invariable sites, four categories of substitution rates). Bootstrap values (1000 replicates) higher 70% are marked at the relevant branches. Phylotypes obtained in this study in bold red. Scale bars represent 1% estimated base substitution.

The two dominant SMAR chemoautotrophic phylotypes were chosen for phylogenetic reconstruction (Fig. 6A). The ML analysis positioned them in a monophyletic clade together with the chemoautotrophic symbionts of *B. azoricus* and *B. puteoserpentis* from the NMAR, *B. brooksi* from the Gulf of Mexico, *B. thermophilus* from EPR, *B. mauritanicus* from the Gulf of Mexico and a species from the Juan de Fuca Ridge. Within this group, the chemoautotrophic SMAR symbionts appeared to be most closely related to those of *Bathymodiolus* sp. from JdF (0.5–0.7% nucleotide divergence), but the phylogenetic relationships were not clearly resolved.

Four clone sequences (two for 5°S, two for 9°S) fell together with methanotrophic γ-proteobacterial symbionts and full sequences were obtained for all four clones. Three of the four sequences were identical revealing a common methanotrophic phylotype in 5°S and 9°S. One sequence from 9°S had two substituted positions (0.17% divergence) which were both located in moderately conserved regions. In the ML analysis, the SMAR symbionts formed a higly supported monophyletic group together with the symbionts of *B. childressi* from GoM and *B. hirtus* from the western Pacific (Fig. 6B). The methanotrophic symbionts of the two NMAR species *B. azoricus* and *B. puteoserpentis* were clearly divergent (2.5–2.7%) indicating that SMAR and NMAR *Bathymodiolus* spp. harbor different methanotrophic phylotypes.

#### Abyssogena southwardae *symbionts*

All 96 16S rRNA clones obtained from the two SMAR *Abyssogena* clams were similar to the chemoautotrophic symbiont of *A. southwardae* from Logatchev. Four out of six full-length sequences (1490–1506 nt) were identical while the two others had two substitutions each (0.17% divergence). These all occurred uniquely and in moderately to highly conserved regions of the 16S rRNA gene alignment, and therefore cannot be distinguished from PCR and sequencing errors. The identical sequences shared 100% identity with a sequence from Logatchev, indicating that the symbionts of *A. southwardae* from Logatchev and 5°S share a common phylotype (Fig. 6C). This common MAR phylotype was clearly divergent from the *A. southwardae* symbiont from western Florida (1.4%).

## Discussion

Our analyses of *Bathymodiolus* and *Abyssogena* hosts and symbionts on the MAR revealed four main results: (*i*) *Bathymodiolus* from the SMAR and the NMAR species *B. azoricus* and *B. puteoserpentis* are monophyletic indicating that their radiation happened recently and after divergence from their sister group of the north Atlantic *B. boomerang* complex. (*ii*) Chemoautotrophic and methanotrophic symbionts of SMAR *Bathymodiolus* spp. are divergent from the *B. azoricus*/*B. puteoserpentis* symbionts. (*iii*) *Bathymodiolus* symbionts do not reflect the phylogenetic relationships of their host species. (*iv*) Identical *Abyssogena southwardae* COI haplotypes in NMAR and SMAR coincide with identical 16S rRNA phylotypes of their symbionts.

### Phylogenetic Relationships of MAR *Bathymodiolus* spp

The monophyly of the SMAR and NMAR *Bathymodiolus* based on the COI gene indicates that all four lineages share the same evolutionary ancestor. This is in contrast to an earlier study of mitochondrial NADH dehydrogenase subunit 4 gene (ND4) suggesting that the *B. boomerang* complex species *B. heckerae* may have derived from *B. azoricus* [35]. That scenario was not well supported by bootstrap statistics, while a recent phylogeny based on COI [15] and our results based on the COI phylogeny, genetic distances and the distribution of non-synonymous substitutions of this gene suggest divergence of the *B. boomerang* complex from the MAR species before these diversified.

*Bathymodiolus* sp. collected at 5°S were at first glance morphologically similar to *B. puteoserpentis* from NMAR. Preliminary examinations of shell morphologies confirmed affinities between 5°S mussels and the Logatchev morphotype of *B. puteoserpentis* (R. von Cosel, pers. information) and 5°S animals were tentatively referred to as *B. puteoserpentis* in a first description of the 5°S vents [18] while the 9°S population shared morphological characteristics with *B. azoricus* (R. von Cosel, pers. information). In contrast to the similarity of the shell morphologies, we measured considerably high genetic divergence between all four MAR lineages and our results suggest a closer relationship of *Bathymodiolus* sp. 5°S to *B. azoricus* than to *B. puteoserpentis*. Even though the COI gene can show a high degree of polymorphism within populations of hydrothermal vent species [10] and we analyzed a limited number of specimens at each SMAR sampling site, the geographic segregation of haplotype groups, the levels of population differentiation between these groups and the measured genetic distances indicate a clear geographic separation of all four MAR lineages. The rate of nucleotide substitutions and genetic distances (Table 2) match or even exceed what was measured for the two East Pacific Rise populations of *B. thermophilus* and *B.* aff. *thermophilus* separated by the Easter Island Microplate, which are considered to represent a recent speciation event [9]. It is possible that the speciation process among SMAR and NMAR *Bathymodiolus* has reached a similar level as in EPR *Bathymodiolus* around the Easter Island Microplate, however, the resolution of the COI gene alone is not sufficient to clarify this (see below).

Our data clearly show a monophyletic relationship among the four MAR lineages and closest K2P-distance between 5°S and 9°S suggests most recent divergence of these two. If the equatorial belt is a dispersal barrier, we would expect a larger genetic distance between southern and northern lineages than within the north or the south. However, the largest distance was between the two NMAR species *B. azoricus* and *B. puteoserpentis* (Table 2). This could suggest that *B. azoricus* and *B. puteoserpentis* diverged before SMAR and NMAR lineages, and in this case, 5°S and 9°S lineages should share a common ancestor with one of the NMAR species. Slightly larger K2P distance and higher number of fixed substitutions between *B. azoricus* and *B. puteoserpentis* than between any of the other three lineages might suggest that *B. azoricus* diverged early from a common ancestor of the other three lineages. On the other hand, the real genetic divergence between pairs of lineages may be obscured by multiple substitutions of synonymous sites. Such substitutions do not affect the amino acid sequence and they can rapidly accumulate in the mitochondrial COI gene [36]. If multiple substitutions occur, the measured genetic distance underestimates the real genetic divergence, in particular if reverse substitutions lead to restorations of ancestral states, but the phylogenetic reconstruction could not resolve the history of their diversification. A reliable phylogenetic reconstruction of MAR lineages therefore requires additional markers.

The two species *B. azoricus* and *B. puteoserpentis* illustrate the complexity of MAR *Bathymodiolus* diversification. Allozymes and multi-locus analyses showed that these two species hybridize in a zone where their distribution ranges overlap, and that asymmetric gene flow mainly from *B. azoricus* to *B. puteoserpentis* is apparent even between geographically distant populations of the two species [37], [38], [39], [40]. Faure et al. (2009) [40] highlighted in a multi-locus population genetic study that it is difficult to decide whether gene flow between these two species happened during parapatric speciation or in the course of secondary contact after a period of allopatry. In the case of secondary contact, it could be possible that divergence of *B. azoricus* and *B. puteoserpentis* started early and that the two species entered the MAR independently at different times [40]. Because of the close relationship between all MAR *Bathymodiolus* lineages, their diversification and also their history of MAR colonization may only be solved by including the SMAR species in future multi-locus analyses using mitochondrial and nuclear genes.

### Dispersal of *Bathymodiolus* spp. along the MAR

Three alternative models for the dispersal of *Bathymodiolus* on the MAR could explain the current species distribution: (*i*) The NMAR as well as both sides of the northern Atlantic Ocean may have been colonized by ancestors of the monophyletic clade of the *B. boomerang* complex and their MAR sister species via the southern Atlantic; (*ii*) ancestors of the MAR species dispersed in seep regions on the American continental margin between the GoM and off Brazil and entered the MAR independently along a longitudinal gradient north and south of the large equatorial transform faults; (*iii*) *Bathymodiolus* from hydrocarbon seeps on the northern Atlantic margins arrived on the northern MAR and dispersed southwards across the equator.

The first model assumes ancestral invasion to the southern MAR via the Indian Ridge and northwards dispersal across the equatorial belt. This is based on the monophyletic relationship of species from the MAR and the Indian and western Pacific Oceans based on mitochondrial COI and ND4 genes [41] and our own COI ML analysis (Fig. 5A). This model would match with a suggested habitat change from vents to seeps in the course of a supposed recent separation of *B. heckerae* from *B. azoricus* [35]. However, low statistical support in our analysis questions the placement of the Indo-Pacific group within the *B. thermophilus* clade. Moreover, if ancestral *Bathymodiolus* invaded the MAR from the Indian Ridge, dispersed northwards and colonized the Atlantic seeps from the MAR, we would expect that the *B. boomerang* complex diverged after the beginning of the MAR species radiation. However, previous analyses by Olu Le-Roy et al. (2007) [15] and our own data indicate the exact opposite, that MAR species radiated after the divergence from the *B. boomerang* species complex. We therefore consider this model the least likely.

The second model assumes that ancestors of the SMAR species dispersed southwards along the American continental margin. This would match a model of deep water currents presented by Van Dover et al. (2002) [1] according to which a second deep-water passage crosses the MAR south of the equatorial belt at 20°S connecting the Atlantic margins off Brazil and Namibia. Such a dispersal path is possible but appears unlikely because in that case we would expect to find species on the American continental margin that are more closely related to SMAR *Bathymodiolus* spp. than the *B. boomerang* complex. Such species have not been discovered: The seeps from the GoM, Blake Ridge and the Caribbean harbor species from the *B. boomerang* complex as well as the more distantly related species *B. brooksi* and mussels from the “*B*”. *childressi* complex [e.g. 15,16,42,43]. The genus *Bathymodiolus* has not yet been detected south of the Barbados seeps and, hence, there is no current support for the independent colonization of the MAR south of the equator.

The third model is the most likely explanation. It matches a current hypothesis according to which *B. azoricus* and *B. puteoserpentis* or a common ancestor entered the MAR from western Atlantic hydrocarbon seeps [37], [40] and it requires an equatorial belt open for dispersal into the southern Atlantic Ocean by west-to-east passage through the long equatorial transform faults. Faure et al. (2009) [40] estimated that *B. azoricus* and *B. puteoserpentis* diversified approximately 0.76 Ma ago, and Miyazaki et al. (2010) estimated that the monophyletic Atlantic species group including the *B. boomerang* complex, *B. azoricus* and *B. puteoserpentis* diversified approximately 6.2 Ma ago [41]. The entrance of *Bathymodiolus* from the western Atlantic margin to the MAR must therefore have happened within this time frame. The closest distance between continental margin seeps and the northern MAR today is 1400 km between the Barbados Accretionary Prism and north of the Fifteen-Twenty Fracture Zone. Assuming a constant spreading rate in this region of 23.5 km Ma^-1^ over the past 60 Ma since the opening of the Atlantic Ocean, the Barbados seeps and the MAR must have been separated by some 1250 km when the first *Bathymodiolus* species arrived. This is significantly more than the 935 km length of the Romanche Transform Fault today, and if west-to-east currents transported *Bathymodiolus* from the American continental margin to the MAR, passage of larvae driven by favorable currents through the Romanche Transform Fault that was even shorter in the past than today is also feasible.

### Gene Flow in *Abyssogena Southwardae*

Our result of a common COI haplotype in *Abyssogena southwardae* in Logatchev and 5°S and very similar haplotypes at MAR sites and the Western Florida Escarpment in the Gulf of Mexico [33], [44] strongly suggest gene flow over large distances. Long-range dispersal capabilities of this species have also been evidenced by morphology data indicating a distribution over wide areas of the North Atlantic from offshore Virginia to the Barbados Accretionary Prism, Logatchev, Vema Transform Fault, 5°S on the SMAR and the Canary Islands [20]. All these locations are widely separated, cover an extensive depth range of 737–5107 m and include vents and seeps. This wide distribution is remarkable because the potential for dispersal in chemosynthetic vesicomyids has been considered limited as inferred from their lecitotrophic larvae which are classically considered short-lived and thus unable to reach remote areas such as widely interspersed hydrothermal vents on mid-oceanic ridges [45]. However, the dispersal capabilities of vesicomyid clams may be much greater, in particular because lecitotrophy may be an advantage for long-range dispersal in the oligotrophic deep sea waters [46]. While the majority of chemosynthetic vesicomyid species is found at continental margins where hydrocarbon seeps are abundant, only a few species occur at hydrothermal vents on the mid-oceanic ridges [47]. Among these, “*Calyptogena*” *magnifica* also shows long-range dispersal by a distribution over more than 4000 km between 21°N and 17°S on the EPR [10]. Further indications for long-range dispersal capabilities of chemosynthetic vesicomyids are given by close phylogenetic relationships based on COI between species pairs from geographically distant sites on the western Pacific and eastern Pacific margins suggesting that ancestral species migrated over large distances [48].

### Biogeography of the Symbionts

#### Bathymodiolus *symbionts*

The phylogenies of MAR *Bathymodiolus* hosts and symbionts are not congruent. Both *Bathymodiolus* lineages from 5°S and 9°S share very similar chemoautotrophic and methanotrophic symbionts, and this similarity parallels previous observations in *B. azoricus* and *B. puteoserpentis* which also share highly similar to identical chemoautotrophic and methanotrophic 16S rRNA phylotypes [34]. In contrast, we observed considerable sequence divergences of 1% for the chemoautotrophs and 2.5% for the methanotrophs between NMAR and SMAR populations, which at first glance does not support genetic connectivity between NMAR and SMAR symbionts. In particular the closest phylogenetic relationship between methanotrophic symbionts of SMAR *Bathymodiolus* and *B. childressi* from the GoM suggests that transport of symbionts from the GoM to the SMAR may have occurred. This would support the hypothesis of an existing west-east passage for chemosynthetic organisms in the equatorial Atlantic [1], [13].

*Bathymodiolus* symbionts are most likely acquired horizontally by uptake from the environment [e.g. 49,50,51,52]. Petersen et al. (2010) [23] discussed two alternative models that could explain the geographical structuring of horizontally transmitted ectosymbionts of the MAR vent shrimp *Rimicaris exoculata*, which may also be valid in *Bathymodiolus* symbioses. In the first model (*i*), barriers limit the dispersal of free-living symbionts leading to geographically isolated bacterial populations. If this was the case in MAR *Bathymodiolus* symbioses, it would mean that SMAR hydrothermal vents provide different populations of free-living symbionts than NMAR vents. An alternative model (*ii*) assumes that dispersal of symbionts is not limited and the free-living populations occur ubiquitously at geographically distinct vents. In this case, geographic structuring of symbiotic associations would be due to specific bacteria-host selection from a pool of diverse free-living forms. The first model requires flexible patterns of host-symbiont recognition when host species colonize new vent sites, while the second model requires highly specific recognition mechanisms between the symbiotic partners. Such highly specific recognition patterns are known from other symbioses with horizontal symbiont transmission [53], [54] but have not yet been identified in *Bathymodiolus*. An additional alternative (*iii*) assumes that dispersing larvae may transport bacteria from their natal sites and “inoculate” new colonization sites with the symbionts of the parental host population or possibly also other bacteria from the parental habitat [51].

There are two explanations for the closer phylogenetic relationships between methanotrophic symbionts of SMAR *Bathymodiolus*, *B. childressi* from GoM and *B. hirtus* from the western Pacific than between SMAR and NMAR. The first is that methanotrophic bacteria closely related to NMAR symbionts were absent in SMAR vents when *Bathymodiolus* invaded from the north (model *i*), and that the hosts established a symbiotic association with a methanotroph at SMAR vents that originated from the GoM. In this first scenario we would assume stronger limits for the dispersal of bacteria along the MAR than for mussel larvae. This seems unlikely, and in fact the opposite is the case for symbionts of *B. azoricus* and *B. puteoserpentis* of the NMAR. Identical phylotypes of the two chemoautotrophic and methanotrophic symbionts co-occur in the two geographically separated mussel species, indicating that both symbionts have a wider distribution than their hosts.

Alternatively, it is possible that SMAR *Bathymodiolus* were initially associated with methanotrophic symbionts closely related to the NMAR symbionts, and that these hypothetical ancestors were displaced by methanotrophic symbionts that originated from the GoM. This would be consistent with larval colonization of sites that provide ubiquitously distributed symbionts (*ii*) and with simultaneous colonization of the sites by larvae and symbionts (*iii*). Displacement of one symbiont by another in marine chemosynthetic symbioses has, to our knowledge, not yet been shown. A possible scenario for how such a displacement event could occur was recently described in the hydrocarbon seep mussel *Bathymodiolus heckerae* that harbors two closely related γ-proteobacterial sulfur-oxidizing phylotypes [55]. Both symbionts were only rarely found in the same bacteriocyte suggesting competition between the two symbionts for the same sulfur sources [55]. If two symbionts in one host use the same source for metabolism, the less competitive one could eventually be displaced. It is possible that after the colonization of SMAR vents ancestors of methanotrophic NMAR and GoM symbionts co-occurred for a while in SMAR *Bathymodiolus*, and that competition between the two for methane eventually lead to the exclusion of the NMAR related symbiont.

#### Abyssogena southwardae *symbionts*

The presence of an identical symbiotic 16S rRNA phylotype in *A. southwardae* from Logatchev and SMAR mirrors the identical COI haplotypes of their hosts. This is in accordance with coupled dispersal of vesicomyid larvae and their symbionts in consequence of maternal (vertical) co-transmission of mitochondria and symbionts across host generations [10], [56], [57], [58], [59] and it is a strong argument for gene flow in clams and their symbionts along the MAR and across the equatorial belt. However, the presence of a divergent symbiotic phylotype in *A. southwardae* from western Florida is surprising. Recently discovered co-occurrence of two unrelated symbiotic phylotypes in a vesicomyid species from the Juan de Fuca Ridge indicated that vesicomyid clams can also acquire symbionts horizontally [60]. Acquisition of the divergent phylotype from the environment appeared more likely than horizontal exchange between co-occurring species or paternal transfer of symbionts associated with genetic hybridization. Divergent symbiotic phylotypes in geographically distant *A. southwardae* specimens could therefore suggest that populations from western Florida or the MAR acquired symbionts horizontally in the past, and would thus represent a second example of non-strictly maternal symbiont transfer in vesicomyids. If horizontal acquisition was in a MAR population, it must have happened before dispersal along the ridge axis, but more solid explanations for the divergence of symbionts in this host species requires more genetic data from these and other *A. southwardae* populations.

### Other Indications for Gene Flow Across the Equatorial Belt

The state of taxonomic analyses of the SMAR collections does not yet allow community analyses, but a considerable degree of similarity between northern and southern MAR vent fauna is currently apparent. This suggests that along-axis faunal dispersal across the equator has happened or may still be happening. The hydrothermal vent shrimp *Rimicaris exoculata* is present at all 5°S hot vents [18]. Common COI haplotypes among the vent sites Rainbow, TAG, Snake Pit, Ashadze, Logatchev and 5°S indicated conspecificity for all MAR *Rimicaris* and recent gene flow across the equatorial belt [23], [61]. Similarly, although their γ-proteobacterial and ε-proteobacterial ectosymbionts showed geographic clustering based on 16S rRNA phylogeny, the genetic differences between these populations were correlated with distance along the MAR and did not indicate a major barrier for gene flow in the equatorial region [23]. The polychaete species *Laonice athecata, Prionospio unilamellata,* and *Amathys lutzi* found at the 5°S and 9°S vents (B. Ebbe, pers. communication) occur also at northern MAR vents. These observations suggest that the SMAR vent sites at 5°S and 9°S are not disconnected from the NMAR.

### Conclusions

Our results show clearly that *Bathymodiolus* mussels and *Abyssogena southwardae* clams from the southern Mid-Atlantic Ridge at 5°S and 9°S are more closely related to species from the northern MAR than to other species: SMAR mussels form a monophyletic clade together with *B. azoricus* and *B. puteoserpentis* while *A. southwardae* from 5°S and from the Logatchev vent field share an identical COI haplotype. This indicates that gene flow between the northern and southern Mid-Atlantic Ridge has happened and may still continue. Our results therefore do not justify a strict dispersal barrier that disconnects northern and southern MAR communities biogeographically. Contrary to previous hypotheses, we did not observe close relationship to Indian Ocean taxa.

The dispersal of *Bathymodiolus* spp. most likely occurred from north to south along the MAR rather than independent colonization events from continental margin seeps north and south of the equator. The distribution pattern of the hydrothermal vent species *Rimicaris exoculata* across the equatorial belt testifies that such a pathway is open to species with high potential for long-range dispersal. *Bathymodiolus* have long-lived planktotrophic larvae that can probably spend up to a year in the water column [62] suggesting that favorable currents may transport them for several hundreds of kilometers. Vesicomyid clams may have much better dispersal capabilities than previously inferred from their mode of larval development, in particular because lecitotrophy may be of advantage for long-range dispersal in oligotrophic deep-sea waters [46]. Strong currents from west to east through the large equatorial transform faults may bridge long distances for larval transport between ridge segments and other chemosynthetic habitats including hydrocarbon seeps and possibly also sunken wood and whale carcasses [63], [64], [65] may serve as stepping stones for hydrothermal vent species. The suitability of an off-axis habitat with fluid flow induced by serpentinization processes was shown for a vent *Bathymodiolus* species at the Lost City site [66], [67], [68]. Other off-axis habitats may also be associated with hydrothermal fluid flow, as evidenced for example in pull-apart basins adjacent to transform faults [69], [70]. The occurrence of *A. southwardae* in the Vema Transform Fault [20], [71] confirmed that such stepping stones exist in the Atlantic.

Our investigations of chemosynthetic bivalves and their symbionts represent an initial test for a dispersal barrier effect of the equatorial belt based on mitochondrial COI and bacterial 16S rRNA marker genes. The results indicate that although host populations are geographically separated, a strict dispersal barrier between north and south does not exist for the species we investigated. Future multi-locus investigations of NMAR and SMAR animals using mitochondrial and nuclear phylogenetic markers will reveal if the equatorial belt exhibits a structuring effect beyond geographic distance on gene flow and speciation processes among MAR populations.
